# Clinical Lectures on Diseases of the Eye

**Published:** 1864-10

**Authors:** E. L. Holmes

**Affiliations:** Chicago, Lecturer on Diseases of the Eye and Ear in Rush Medical College, and Surgeon of the Chicago Charitable Eye and Ear Infirmary


### CLINICAL LECTURES ON DISEASES OF THE EYE.

By E. L. HOLMES, M. D., of Chicago,
*Lecturer on Diseases of the Eye and Ear in Rush Medical College, and Surgeon of the Chicago Charitable Eye and Ear Infirmary.*

### THE IRIS.—ANATOMY AND PHYSIOLOGY.

Gentlemen :—We have, thus far in our course, finished a brief examination of the conjunctiva, cornea, and their diseases. I need scarcely repeat that these diseases are the most important for you to understand, since they are the most common, and, if neglected, perhaps the most destructive to vision.

In the limited time to which we have been confined, I have necessarily been unable to present to your consideration more than the outlines of the subject—to teach you how to study these diseases by showing you as many cases as possible, and by referring you to the most reliable authorities.

We now come to an entirely different series of structures, the Iris, Crystaline Lens, and Ciliary Processes—the study of their anatomy, physiology, and their diseases, which are of uncommon interest. As heretofore, I shall necessarily be brief and present you but the outlines of the subject I think you will not fail to follow with interest.

The Iris is a thin, circular membrane, composed of areola tissue, containing muscular fibres, vessels and nerves. This areola tissue is filled with a number of star and spindle shaped cells, with minute appendages, anastomosing with each other, as described in the lecture upon the conjunctiva. These cells are, to a greater or less extent, supplied with pigment.

The muscular fibres are non-striated and are arranged in two distinct groups; the circular running parallel with the pupil in concentric rings; and the straight, radiating fibres, which are arranged between the circumference of the iris and the pupil, like the spokes of a wheel. The contraction of the former muscles diminishes the size of the pupil, that of the latter increases it.

Although numerous pigment cells are found throughout the whole substance of the iris, they are especially abundant in its posterior layers. The amount of pigment in this layer is perhaps more uniform in different individuals than in the other layers. In individuals with light-colored irides this pigment shows through the other layers which are comparatively free from pigment, giving a blue or gray color to the eyes. Infants usually have but little pigment in the anterior layers of the iris, hence their eyes are generally blue or gray. As they become older, more pigment is deposited. The different shades of color in the iris of the adult depends upon the amount of pigment in the anterior layers of the iris. Occasionally pigment is so unequally distributed in the iris that small dark spots are observed upon its surface.

The posterior surface of the iris is covered with minute radiating lines, continued from the ciliary processes. The whole iris is covered with a delicate epithelial layer.

The diameter of the iris is more than a line longer than that of the cornea. This is an important point in connection with the operation for artificial pupil. An instrument introduced through the sclerotic three-fourths of a line behind its union with the cornea, if held parallel with the plane of the iris, will enter the anterior chamber without injuring the iris.

The iris is attached at its periphery to the cornea and ciliary processes. This attachment is quite delicate, and seems to vary in strength in different individuals, if we may judge from the readiness with which it is broken by slight blows upon the eye. The iris is hung like a curtain in the space between the lens and the cornea, dividing it into two chambers, the anterior and posterior. The papillary edge of the iris is in contact with the anterior surface of the capsule of the lens. The posterior chamber is therefore very much smaller than the anterior, being in fact but a very narrow space near the periphery of the iris.

The iris is supplied with nerves of sensation from the fifth pair; the motor nerves of the circular muscles are derived from the third pair, and the motor nerves of the radiating fibres are derived from the sympathetic. These nerves, after passing from the ciliary ganglion, situated just behind the eye at the outside of the optic nerve, divide into twelve or more filiments, which enter the sclerotic and pass forward in the choroid to the ciliary muscle and iris.

The vessels of the iris are the ciliary arteries and veins—branches of the ophthalmic artery and vein.

The functions of the iris are three-fold: to regulate the amount of light which enters the eye; to secrete the aqueous humor; and to assist indirectly in adjusting the eye for vision at different distances.

Normal changes in the size of the pupil are produced by variations in the amount of light, which falls upon the retina. A ray of light shining upon a portion of the iris above, without passing through the pupil, will not cause it to contract. But if the ray enters the pupil, the contraction occurs at once.

Although the iris is believed by most physiologists to be the organ which secretes the acqueous humor, it is evidently not the sole source of this fluid, since it is still found in the anterior chamber, when the iris has been removed from the eye by accident. It is so richly supplied with blood and secretes the acqueous humor with such rapidity, that when wholly evacuated through an incision in the carnea the anterior chamber is refilled in a couple of minutes.

When we come to speak of the power of adjustment of the eye for vision at different distances, your attention will be called to the part performed by the iris in this act.

There are several interesting and important points regarding the pupil which you should remember, not only as students of diseases of the eye, but as students of general Medicine. In examining an eye with a dilated pupil, you should bear in mind that the patient may have used, with or without design, Belladonna in some form. With the pupil contracted there might be reason to inquire whether Calabar Bean had been employed.

In examining an eye in reference to the movability of the pupil, the other eye should be kept covered, while the former should be alternately covered and uncovered with the hand. The following fact will explain the reason why this should be done: If both eyes are exposed to light, on covering only one of them the pupil will dilate but half as much as it would if the other was covered also. The manner I have just described will enable you to obtain the greatest possible change in the size of the pupil.

The pupil is much contracted during sleep. The iris resembles the other sphincters of the body in being thus firmly contracted during sleep.

The condition of the pupil is an important symptom in several pathological changes in the nervous system. A constantly dilated pupil is often a symptom of disease of the optic nerve, so often called amaurosis. Sometimes, however, when a patient cannot distinguish day from night, the pupils will dilate and contract upon alternately exposing the eyes to light and shade.

Contraction of the pupil often occurs when the ocular branches of the fifth pair of nerves are irritated, as for instance by the presence of a mote upon the cornea or under the lid.

Whatever produces directly or indirectly either irritation of the nerves supplying the circular fibers of the iris, or paralysis of those supplying the radiating fibers, will cause contraction of the pupil. But either paralysis of the circular or irritation of the radiating muscles will produce dilatation of the pupil. Certain fractures of the skull, compression of the brain, tumors, and effusions of blood or serum in the brain, by paralyzing the nerves of the circular fibers often produce dilatations of the pupil. Irritation of the bowels, onanism, seem to produce the same condition of the iris by irritation of the nerves of the radiating muscles.

Belladonna appears not only to paralyze the circular muscles, but also to excite the radiating fibers to contract, thus producing dilatation of the pupils. When taken internally this drug passes by the circulation to the nervous centres. When applied locally it acts directly upon the iris itself, being absorbed by the tissues and conveyed directly to the iris.

It is perhaps worthy of notice that the pupils are contracted when the axis of the eyes are convergent, as in looking at near objects. On the other hand, they are dilated when the axes are divergent, as in looking at distant objects.

In near-sighted individuals the pupils are usually dilated and situated quite far behind the cornea. On account of this great depth of the anterior chamber the cornea appears more convex than it really is.

In far-sightedness the pupil is usually much contracted and placed forward with the lines quite near the cornea; the anterior chamber is consequently of comparatively little depth.

These subjects will be more fully discussed in a subsequent lecture.

